# Machine Learning-Based Surrogate Modelling for Efficient Inverse Analysis of Micro-Indentation Response to Determine Material Parameters

**DOI:** 10.3390/ma19122435

**Published:** 2026-06-07

**Authors:** Sidrah Sajjad, Sebastian Knorr, Dirk Schellenberg, Thomas Chudoba, André Clausner, Alexander Hartmaier

**Affiliations:** 1 Interdisciplinary Centre for Advanced Material Simulation (ICAMS), Ruhr-Universität Bochum, Universitätsstr 150, 44801 Bochum, Germany; alexander.hartmaier@icams.rub.de; 2 SENGICON GmbH, 09116 Chemnitz, Germany; sebastian.knorr@sengicon.de (S.K.); dirk.schellenberg@sengicon.de (D.S.); 3 ASMEC Advanced Surface Mechanics GmbH, 01109 Dresden, Germany; t.chudoba@asmec.de; 4 Fraunhofer IKTs, 01109 Dresden, Germany; andre.clausner@ikts.fraunhofer.de

**Keywords:** micro-indentation, surrogate modelling, numerical database, optimization, inverse problem, uniqueness

## Abstract

Inverse analysis from indentation experiments has been a challenging problem due to the nonlinear relationship between indentation response and material parameters. In this work, a data-driven method is proposed that integrates an artificial neural network (ANN) and evolutionary optimization for the reliable and efficient inverse parameter identification. A large dataset is generated by simulating the indentation process based on different combinations of material parameters in a systematic way. Then, by using the simulated data, a set of ANN models is trained that can efficiently predict the indentation responses, i.e., the displacement–time curve, the indentation force, and the surface profile, as a function of material parameters. These trained models exhibit the potential to replace the computationally expensive numerical simulations for the identification of material parameters by inverse analysis. In this way, the surrogate models make the numerical evaluation of the loss function, which is minimized during the inverse analysis, orders of magnitude faster. This enables the use of the powerful genetic algorithm for the minimization of the loss function, which would be impossible without numerically efficient surrogate models, as this algorithm requires many iterations to produce robust results. In this work, we systematically investigate which mathematical loss function leads to robust and unique results in determining the material parameters through inverse analysis of indentation results. The results show that such an inverse analysis can be successfully performed for simulation data. In forthcoming work, this method will be generalized to experimental indentation data, which will allow the characterization of the mechanical behaviour of materials by micro- or nano-indentation tests.

## 1. Introduction

Over the years, various techniques have been investigated to efficiently assess the mechanical properties of materials, including stiffness, yield strength, ultimate tensile strength, toughness, and ductility. Tensile testing and indentation methods [1] are the most commonly used approaches to determine the material parameters that describe its response to applied mechanical loads. Although the tensile test is considered the gold standard for evaluating the complete stress–strain curve, it also has some practical limitations due to the complex equipment, substantial sample size, and repeated measurement requirements. As an alternative approach, instrumented indentation has emerged as a quasi-non-destructive method allowing comparatively simple sample preparation and testing. This method can characterize the mechanical properties by continuously recording force–displacement data during indentation [2,3]. Conventionally, indentation tests result merely in a hardness value for the material, which is of limited value compared to a tensile test. In recent years, several groups have demonstrated that the mechanical behaviour of materials can be estimated by the inverse analysis of indentation data, combining experiment and numerical modelling. However, estimating stress–strain curves from indentation response has remained a challenging task due to the indirect nonlinear relation between material parameters and indentation results [4].

In the past decades, the finite element method (FEM) has been extensively used for the inverse analysis of indentation data for predicting the mechanical properties of materials. The basic idea of inverse parameter identification is to use finite element simulations to reproduce the experimental indentation curves by solving a constrained optimization problem for the material parameters used in the model. Many researchers have proposed a finite element-based inverse method for the identification of plastic material properties, e.g., yield strength and work hardening rate, by using the residual imprint profile obtained from sphero-conical indentation [5,6,7]. Sajjad et al. [8] presented a novel hybrid method to identify the kinematic hardening parameters describing cyclic plasticity of metals by combining FE simulations and cyclic Vickers indentation. They also did the comparison of the J2-plasticity and crystal plasticity by implementing the Chaboche kinematic hardening model to capture the cyclic hardening behaviour of the materials. Furthermore, in another investigation [9], the comparison of one-step and two-step optimization strategies is drawn to identify the material parameters for time-dependent viscoplastic and work hardening behaviour of the material. Frydrych and Papanikolaou [10] proposed that the best way is to fit both the load–displacement curve and the imprint profile at the same time for the unambiguous identification of material parameters. Clyne et al. [11] reviewed a profilometry-based inverse approach that couples the indent profile from the spherical indentation test and the FEM simulation to extract the true stress–strain behaviour in the plastic regime. In another research [12], Wang et al. proposed a Bayesian inverse framework to estimate the elasto–plastic material parameters from spherical indentation experiments. They used proper orthogonal decomposition (POD) to efficiently use the imprint profile for the identification of material parameters. They also proved that the non-uniqueness of parameters improves by incorporating multiple indentation loads. Further work [12,13,14] also covered the challenges related to the optimizations, experiments, and FEM simulations. Furthermore, the sample-related problems, e.g., residual stress, anisotropy, and inhomogeneities, are also addressed in their study. In the field of indentation testing, Olaf and Sommer [15] also contributed by investigating the influence of geometrical imperfections of the indenter on the material behaviour by using experiments and simulations. They did this investigation by comparing both the coated and uncoated materials. Despite the several key advantages of FE-based inverse parameter identification and reliable results, the iterative optimization procedure requires repeated FE simulations, which cause high computational costs, due to significant mesh requirements and the need to solve a contact problem. This limits the choice of optimization methods very significantly to a few efficient ones that approximate a minimum of the loss function with a rather small number of iterations, for example the Nelder–Mead method or the Trust-Region approach with constraints.

With the advancement in data-driven methods, surrogate models have emerged as an efficient alternative to finite element simulations. These surrogate models have the potential to reduce the computational expenses to solve a constrained optimization problem [16] and provide the statistical approximation of the complex nonlinear behaviour of materials or even the flexural performance of entire structures [17,18]. Once the surrogate models are trained on the simulation-based dataset, they can rapidly predict even complex relationships between the input and output without the need for repeated finite element simulations. Over the past few years, neural network-based models have been widely acknowledged as a mainstream tool to represent the nonlinear mapping of the inverse problem and high-dimensional inputs effectively [19]. Among various neural networks, artificial neural networks (ANNs), also known as multiple-layer perceptrons (MLPs), have been extensively used to solve inverse indentation problems with improved accuracy and robustness. The ANN consists of neurons arranged in an architecture consisting of input, output, and hidden layers interconnected to each other by nonlinear functions and weights, which process information in a way inspired by the human brain [20,21]. This architecture can be controlled according to the desired inputs and outputs by adjusting the hyperparameters during the training process [22]. The back propagation algorithm is responsible for the modification of the weight connections between neurons during training, based on the error between the predicted and target values [19]. In recent years, numerous scientists have demonstrated that neural networks can implicitly encode the underlying physics of the indentation process. In a study, Jiao et al. [23] applied a neural network (NN) to extract the accurate stress–strain curves purely from the pile-up and applied indentation load, without requiring the depth sensing information. They have demonstrated that the NN can efficiently learn the elasto–plastic response of materials just from indentation pile-up. Živković et al. [19] did a comparative study on ANN and multivariate regression analysis (MRA). They found that the ANNs are well suited for solving nonlinear problems and to meet the industrial technical demands. Kim et al. [24] also adopted a combination of FEM-based simulations and an autoencoder (AE)-shaped ANN model to derive the true stress–strain curves from the indentation results. They confirmed that the true stress–strain curves can be reproduced by using even noisy experimental load–displacement curves. Klötzer et al. [25], Tyulyukovskiy and Huber [26] developed a method for the direct identification of viscoplastic material parameters from spherical indentation data. In the first part of their research, they trained a set of neural networks that can identify all unknown material parameters without optimization. In the second part, the robustness of the proposed method was investigated through experiments for validation purposes.

The present study focuses on inverse parameter identification from spherical indentation data by coupling ANN-based surrogate models and optimization methods. A database for the so-called forward problem, i.e., establishing a function between material parameters and indentation results, is generated by finite element simulations covering a pre-defined range of material parameters. Three surrogate models based on ANNs are developed by using a large amount of data, establishing a mapping between the indentation response (maximum indentation force, displacement–time curve, and remaining surface profile) and the representative material parameters. Then, the trained surrogate models, coupled with a powerful optimization algorithm, are employed for inverse identification of material parameters. The performance of the proposed method is evaluated with respect to robustness, accuracy, and computational efficiency. Additionally, the uniqueness of the identified material parameters is analyzed in this work.

## 2. Constitutive Models for Mechanical Behaviour

In this investigation, the Chaboche [27] hardening model, including both nonlinear kinematic hardening and isotropic hardening, is employed to capture the cyclic hardening behaviour of materials. It is a widely used hardening model, capable of describing cyclic plasticity, including hardening, softening, the Bauschinger effect, and ratcheting under stress-controlled loading [28]. In particular, the use of multiple backstress terms, enables the model to reproduce the complex ratcheting phenomena occurring for large stress amplitudes [29,30]. At the same time, it can make the parameter handling more complex. Moreover, adding more than three terms does not show a significant improvement in the predictive capability of the model. The studies [8,30] showed that two backstress terms are sufficient to describe not only the ratcheting behaviour under moderate stress amplitudes but also the stress–strain (σ-ε) relations when the applied alternative stress $\sigma_{a}$ is smaller than the yield strength $\sigma_{y}$ [30]. In the present work, we also use two backstress terms to avoid parameter redundancy, which might cause limited sensitivity and parameter identifiability. The zeros of the yield function, defined as

(1) $$f=\sqrt{\frac{3}{2}\left(S-\alpha^{\prime }\right):(S-\alpha^{\prime })}-R,$$

based on the von Mises yield criterion [31], represent the yield surface. Here, *S* represents the deviatoric stress tensor, $\alpha^{\prime }$ is the backstress tensor indicating the centre of the yield surface, and *R* is the strain-dependent yield resistance of the material. The yielding of material starts once the second deviatoric stress invariant *J*2 reduced by the backstress reaches the yield resistance, which also covers isotropic hardening in the form

(2) $$R=\sigma_{y}+Q\left(1-e^{-b\varepsilon_{eq}}\right),$$

where $\sigma_{y}$ is the initial yield strength, $\varepsilon_{eq}$ is the accumulated plastic strain, b controls the rate of isotropic hardening and Q is the maximum increase in strength.

**Figure 1:**
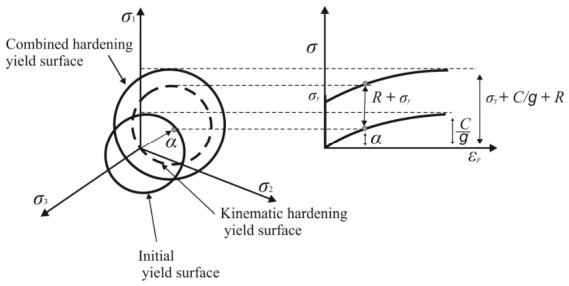
Schematic representation of the combined hardening model depicting the translation of the yield locus by kinematic hardening and its expansion by isotropic hardening; after [27].

The kinematic hardening formulation consists of multiple backstress components, and each backstress term is characterized by two material parameters, *C_i_* and $g_{i}$ as a function of plastic strain, as

(3) $$\alpha =\sum_{i}^{n}\alpha_{i};\;d\alpha_{i}=\frac{2}{3}C_{i}d\varepsilon_{p}-g_{i}\alpha_{i}d\varepsilon_{eq},$$

where *C_i_* and $g_{i}$ control the initial kinematic hardening modulus and its saturation rate, respectively, with respect to plastic strain increment $d\varepsilon_{p}$. Figure 1 illustrates the evolution of the yield surface, including translation and expansion for kinematic and isotropic hardening, respectively.

To simulate the time-dependent plastic flow, the regularized and integrated form of the classical strain-hardening law, known as the strain-hardening power-law creep model [32], is implemented in this work. The mathematical representation of the creep model is

(4) $${\dot{\bar{\varepsilon }}}^{cr}={\dot{\varepsilon }}_{0}\;{\left[{\left(\frac{q}{q_{0}}\right)}^{n}{\left({\left(m+1\right)\;\bar{\varepsilon }}^{cr}\right)}^{m}\right]}^{\frac{1}{m+1}}$$

It represents the equivalent creep strain rate as a function of current relative stress $\frac{q}{q_{0}}$ and accumulated creep strain ${\bar{\varepsilon }}^{cr}$. Here, *q* is the equivalent stress, $q_{0}$ is a reference equivalent stress, ${\dot{\varepsilon }}_{0}$ is the reference strain rate, and *n* and *m* are stress and strain hardening exponents, respectively.

## 3. Forward Model of Indentation Test

### 3.1. Finite Element Modelling of Indentation

**Figure 2:**
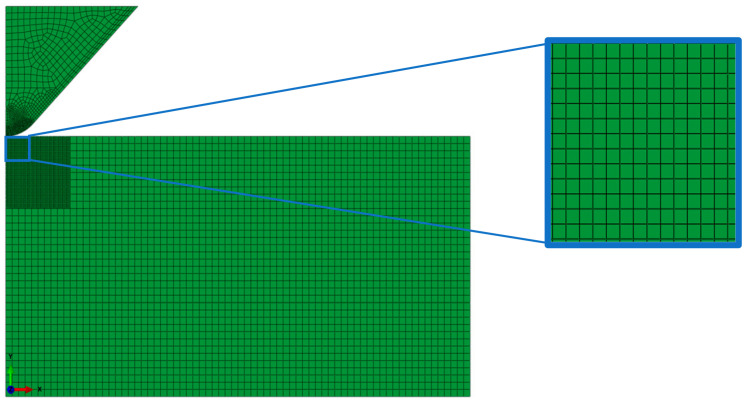
A 2D axisymmetric FE model used to generate indentation data showing two parts: a deformable spherical indenter and a specimen with refine mesh at the contact region.

A two-dimensional (2D) axisymmetric FEM model is developed for the simulation of the indentation process by implementing the commercial software ABAQUS 2022 [32]. The development of this FEM model is inspired by a previous study [9] that validates the 2D and 3D indentation models by calibrating indentation experiments for inverse analysis. Due to the rotational symmetry of the model, only the meridional section is considered, which reduces the numerical calculation cost and yields a comparable accuracy in contact response to that of a three-dimensional model [9]. Figure 2 depicts the geometry of the used 2D axisymmetric FEM model. It consists of a spherical indenter of radius 30 µm, defined as an elastic indenter, and is allowed to move only in the vertical direction. The Young’s modulus and Poisson’s ratio for the elastic indenter are 1100 GPa and 0.11, respectively, mimicking the elastic properties of diamond. The load is applied to the surface of the indenter. The probe is completely fixed at the bottom, and displacement is restricted to the symmetry axis. A surface-to-surface type of interaction is used to establish contact between the indenter and the probe. Based on the study [9], describing the friction effect between a spherical indenter and the specimen, the friction coefficient is set to 0.1 in this work. The entire structure follows the constitutive modelling behaviour described in Section 2. A refined mesh size of 0.5 µm is applied particularly for the contact region of the specimen beneath the indenter, while the coarser mesh is adopted away from the contact area. The type of elements is CAXBR. By following this pattern of discretization, the numerical stability of the simulations is ensured.

### 3.2. Data Generation by FEM Simulations

To generate the dataset for the training of the surrogate forward models, a comprehensive FEM simulation campaign is conducted. In this study, the unknown material parameters comprise the initial yield stress ($\sigma_{y}$), three kinematic hardening parameters ($C_{1}$, $g_{1}$, $C_{2}$), two isotropic hardening parameters (Q,b), and two creep parameters (n,m). In total, there are eight material parameters that need to be identified in this work. All other model parameters are kept constant; in particular, the Young’s modulus and Poisson’s ratio are kept fixed at 211 GPa and 0.3, respectively, for all simulations. The ranges for all variable material parameters that are considered as input variables for the training of the forward surrogate models are given in Table 1. Those parameters cover a rather broad range of material behaviour, which is meant to include in particular steels and other high-strength alloys. At the beginning of the study, each material parameter was assigned to three discrete levels, resulting in a full-factorial design of experiments (DoE), which is employed to systematically explore the entire parameter space, resulting in 3^8^ = 6561 unique combinations of parameters. However, the initial training results indicated that the data on this DoE grid is not sufficient to get reliable surrogate models. Therefore, the dataset has been augmented by approximately 1900 additional off-grid simulations, where the material parameters have been selected randomly on intermediate values between the defined levels of the full-factorial DoE, which significantly improved the performance of the surrogate models.

For each parameter combination, an FEM simulation is performed to compute the corresponding indentation response, which serves as the output. This approach ensures the uniform sampling of the parameter’s input domain, enabling the model to learn the underlying nonlinear relationships between the input parameters and the indentation response. The simulations are performed in two steps. First, displacement-controlled simulations are conducted up to 20% of the indenter radius to extract the maximum indentation force. Then, force-controlled simulations are performed by using the previously extracted force. The following force–time profile is applied to all simulations and comprises one indentation procedure: The indenter is loaded to the maximum indentation force over 10 s and held for 20 s to capture the time-dependent material response. Then it is unloaded to 1% of the maximum force, followed by reloading, a second holding, and a final unloading, where each step has a duration of 5 s. These simulation protocols are based on the indentation experiments, which are the ultimate target of the subsequent investigations.

**Figure 3:**
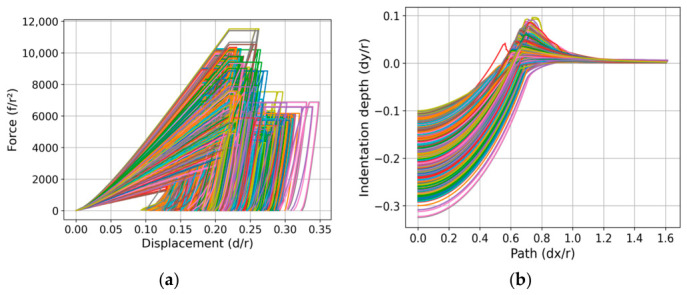
Visualization of the diversity of all indentation responses with different colours and the range of values covered by the generated datasets from FE simulations for a full-factorial design: (**a**) All force–displacement curves depicting both (force–time) and (displacement–time) data scaled with respect to indenter radius. (**b**) All imprint profiles scaled with respect to indenter radius.

The forward database generated by FEM simulations is shown in Figure 3. It visualizes the diversity of the indentation response, e.g., force–displacement curves and imprint profiles for all parameter combinations. Here, all the maximum indentation forces and the displacement–time profiles are also shown by force–displacement curves; see Figure 3a. However, the training data comprise maximum indentation forces, displacement–time curves, and imprint profiles as outputs. Note that all generated data are normalized according to the radius of the spherical indenter, which is 30 μm in this study.

### 3.3. Surrogate Model-Based on Neural Networks

To approximate the FEM response, three forward surrogate models are developed using a multilayer perceptron (MLP). This method is selected after performing a comparative study between the performance of Random Forest Regression (RFR), Support Vector Regression (SVR) and MLP. Among all these methods, MLP consistently provided significantly improved predictions for all developed forward models. In the present work, only the results based on the MLP architecture are presented. The input layer consists of eight input material parameters, and the output layer is formed by the indentation response. All surrogate models are trained using the same inputs, targeting different outputs, namely, maximum indentation force (IF), displacement–time curve (DT), and imprint profile (IP). In the case of DT and IP, the models are trained to predict the entire response curve in a single forward pass. For each model, multiple neurons form the output layer corresponding to their discretized points. The output response for DT is formulated by 250 discretization or sampling points, while the IP is represented by 96 points. Here, the sampling points of IP are truncated beyond the zero-crossing point, as it contains low informative features that can negatively influence the learning of dominant imprint characteristics. The training after truncating all imprint profiles has significantly improved the accuracy of the IP model. The sampling points are equidistant in time for the DT curve and also with respect to the path along the surface for the indentation profile. Concerning the architecture of the ANN, two hidden layers with different numbers of neurons are used to construct the surrogate models for both DT and IP, whereas only one hidden layer is found to be sufficient for the model to predict the IF. The optimal architecture for each surrogate model is selected based on prediction accuracy and generalization capability. For the hyperparameter optimization, an N-fold splitting of the normalized dataset into 80% training and 20% testing subsets is applied. Then, the final training is performed for 100% of the training data. Finally, all trained surrogate models are validated on the same validation dataset consisting of 254 samples generated at random intermediate points between the nodes of the DoE grid. This validation dataset has neither been used for training nor for hyperparameter optimization.

**Figure 4:**
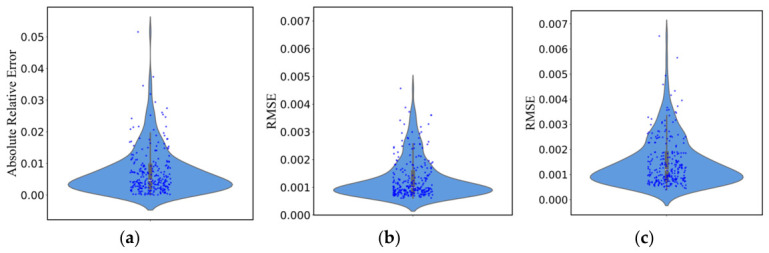
The violin plots show the distribution of predictions error by three trained surrogate models evaluated on 254 unseen off-grid test sets, and each blue point corresponds to an individual test set: (**a**) Surrogate model to predict the maximum force, evaluated using absolute relative error. (**b**) Surrogate model to predict the displacement–time curve, evaluated using RMSE. (**c**) Surrogate model to predict the imprint profile, evaluated using RMSE.

**Figure 5:**
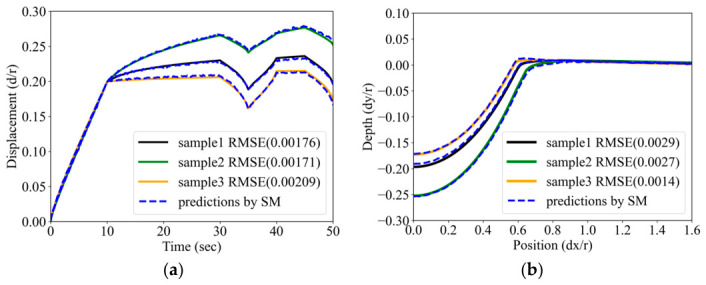
Comparison between three randomly selected FEM-based indentation responses and predictions by their corresponding surrogate models: (**a**) prediction accuracy of displacement–time curves, measured by RMSE, (**b**) prediction accuracy of imprint profile, measured by RMSE.

All surrogate models showed a very good approximation of the FEM results, offering a significant reduction in computational cost. To quantify the accuracy of each trained surrogate model, violin plots are employed to visualize the error distributions at the overall prediction of the IF, DT, and IP, respectively; see Figure 4. The predictive capability of each model is evaluated on the same validation dataset using the different error metrics appropriate to each model’s response. For the prediction of the IF, the model assessment is represented by the absolute relative error between the predicted and reference value of the maximum indentation force. Figure 4a illustrates the absolute relative error distribution with an average of 0.0072. In this case, the maximum relative error of 0.0515 and a median error of 0.0047 are found. This indicates the accuracy of the peak maximum force within an acceptable range across the validation set. In indentation-based inverse modelling, the error between 1 and 5% is acceptable due to the complex nonlinear relationship between material parameters and indentation response. Here, the average relative error of 0.7% is sufficiently low for its practical application. For both DT and IP, the model accuracy is quantified using root mean squared error (RMSE), defined as $\sqrt{\frac{1}{n}\sum_{i=1}^{n}{\left(y_{i}-{\hat{y}}_{i}\right)}^{2}}$, where $y_{i}$ and ${\hat{y}}_{i}$ represent the true and predicted values, respectively and n is the number of data points. The model for DT showed the highest accuracy with a maximum RMSE of 0.0045 and a median error of 0.0009. However, these measurement errors were found to be slightly higher for the imprint profile, which are 0.0056 and 0.0011, respectively. Overall, both models exhibit very good performance, showing an average RMSE of 0.0013; see (Figure 4b,c). The similar results demonstrate that both surrogate models are equally effective and capable of capturing the evolution of both the DT and the IP. To visualize the small remaining discrepancies between the FEM-based indentation response and the predictions by the corresponding surrogate models, only three randomly selected examples are shown in Figure 5 and Table 2.

These samples consist of an off-DoE-grid combination of parameters that are not part of the training dataset. In these examples, the prediction accuracy is measured by following the same error metrics as described above for the violin plots. The surrogate models for DT and IP showed very good comparability between the simulation curves and the predictions by the surrogate models in all three examples. However, a small discrepancy persists depending on the nature of the curves; see Figure 5. Table 2 shows the discrepancy between the numerical IF and the prediction by the surrogate model based on the absolute relative error between them. Among these examples, Sample 2 showed the highest absolute relative error of 0.021, and the lowest absolute relative error of 0.003 is shown by Sample 1. The comparative analysis demonstrates that the maximum indentation force model exhibits a relatively inferior performance compared to the models for curve-based prediction. The variation in performance of surrogate models is due to the different nature of the outputs. The maximum indentation force is a single scalar value, which shows higher sensitivity to the nonlinear relationships with input parameters at the peak of the loading stage; meanwhile, the other two models learned well from the multiple output points, enabling them to capture the characteristics of complete curves more effectively.

## 4. Application of Trained Surrogate Models for Inverse Parameter Identification

### 4.1. Inverse Analysis Framework

**Figure 6:**
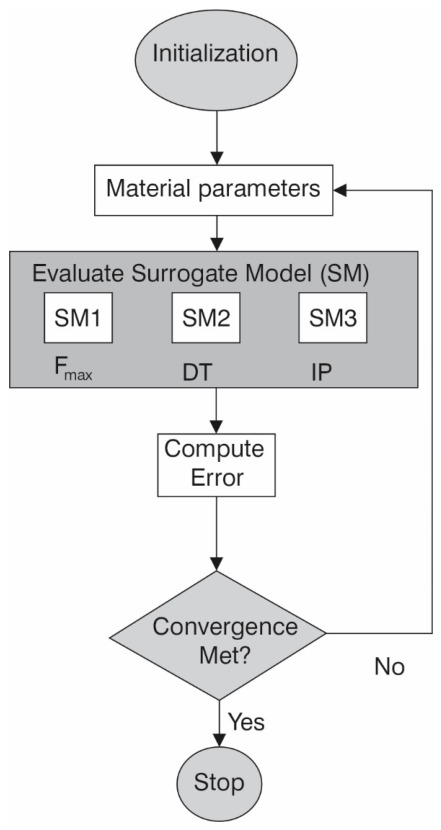
Flow diagram of the proposed framework for inverse analysis, integrating the optimization and three developed surrogate models to predict the indentation responses.

An inverse problem is formulated to reproduce the given indentation response by identifying the unknown material parameters. For the identification of material parameters, the applicability of the trained surrogate models is analyzed by building a framework that combines the developed forward surrogate models with the genetic algorithm (GA). The flow diagram of the developed framework is demonstrated in Figure 6. The three developed surrogate models are described in Section 3. These surrogate models are subsequently employed to estimate the unknown material parameters from their rapid indentation response. A powerful global optimization algorithm, the genetic algorithm, is employed for its robustness in solving nonconvex and nonlinear problems. This algorithm is well suited for the indentation-based inverse problems, where the existence of multiple local minima makes the problem more challenging [33].

To validate the developed framework for inverse analysis under controlled conditions, the synthetic dataset based on FEM simulations is preferred in this study due to the known parameters. These numerical datasets serve as reference targets, enabling a direct comparison between the identified parameters and their actual values. The optimization procedure starts with the random initialization of parameters within their defined bounds. During the whole inverse analysis, the parameter bounds are kept fixed as defined for data generation; see Table 1.

Furthermore, the hyperparameters of the genetic algorithm are kept fixed for all optimizations. The algorithm proposes a candidate parameter set by searching the parameter space iteratively. For each candidate, the trained surrogate models are evaluated to predict the corresponding indentation response, including IF, DT, and IP. To quantify the discrepancy between the target indentation responses and the predictions by the surrogate models, an objective function is evaluated iteratively until the convergence criteria are met, such as reaching the minimum of the function or the maximum number of generations. The parameter set obtained after minimizing the objective function is identified as an optimal solution to the inverse problem.

### 4.2. Objective Function Formulation

For the inverse analysis, it is required to define an objective function over the domain of material parameters. This objective function needs to quantify the discrepancy between the indentation results obtained for any possible choice of material parameters with respect to a fixed reference solution, for which the parameters are known. First, the trained surrogate models are evaluated to predict the corresponding indentation response for a given reference parameter set. Then the discrepancy between the surrogate predictions and FEM simulation results is analyzed. Although the surrogate models are capable of closely approximating the FEM responses, some disagreement between them remains unavoidable. To formulate the objective function, the predictions by the surrogate models are used as reference indentation responses instead of the actual FEM-based response. This approach is adopted to allow the optimization to focus on the convergence instead of compensating the approximation error. In this way, the consistency between the model prediction and the optimization is ensured.

A weighted sum approach is adopted to construct the objective function by combining the error contributions from three surrogate models for maximum indentation force (IF), displacement–time curve (DT), and imprint profile (IP), respectively. To formulate an objective function with appropriate weights, first, the IF, DT, and IP are optimized independently to investigate their individual effect on the identified material parameters. In this investigation, each objective function was defined as the mean absolute error (MAE) between the target indentation responses IF, DT, and IP and the corresponding predictions of the surrogate models. The bounds of parameters for the optimization are the same as those used to design a full-factorial plane to generate the data; see Table 1. During the optimizations, it is observed that the objective function gradually approaches zero. A comparative analysis of optimal solutions derived from each case showed that the optimal parameters obtained from the DT curve and the IP are very close to the reference parameters. However, by optimizing the objective function based on the error in IF, several optimal solutions with a distinct combination of parameters are observed with similar error functions, indicating that the inverse problems based solely on IF are not unique. Based on a comparative study of individual optimal indentation results, a combined objective function considering weighted errors in the individual results for IF, DT, and IP is introduced as

(5) $$f\left(x\right)=f_{DT}\left(x\right)w_{DT}+f_{IP}{(x)w}_{IP}+f_{IF}{(x)w}_{IF}$$

where x denotes the vector of unknown parameters, $f_{DT}$, $f_{IP}$ and $f_{IF}$ represent the mean absolute error (MAE) for DT, IP, and IF, along with their corresponding weights $w_{DT}$, $w_{IP}$ and $w_{IF}$ respectively. In this study, a weighting of 70% to the DT response ($w_{DT}=0.7$) and 30% to the imprint profile ($w_{IP}=0.3$) has been empirically found to give the best results. The weight factor $w_{IF}$ for the indentation force is set to zero, as preliminary results revealed that the inclusion of the maximum indentation force in the objective function yields non-unique solutions. In this way, the whole optimization process is confined to the purely curve-based indentation responses.

## 5. Results of Inverse Analysis

### 5.1. Procedure for Inverse Parameter Identification

To validate the robustness of the proposed inverse analysis framework, twenty-eight target datasets based on high-fidelity FEM simulations, with off-DoE-grid material parameter sets, are analyzed for the convergence stability and the uniqueness of the inversely identified parameters. It is noted that these parameter sets have neither been used for training nor for hyperparameter optimization. The same optimization procedure, bounds, and objective function as described in Section 4 are applied in all cases. Only three examples, selected based on their comparability to the experimentally observed behaviour for martensitic steels, are presented here to describe the detailed identification procedure. However, all twenty-eight samples are statistically analyzed to conclude the study. The selected three reference parameter sets are depicted in Table 3, Table 4 and Table 5.

To investigate the reproducibility of the inversely identified parameters, five independent optimizations are conducted in each case to compare the found solutions with the reference parameters. Here, one solution consists of eight identified material parameters ($\sigma_{y}$, $C_{1}$, $g_{1}$, $C_{2}$, Q, b, n, m) for each optimization. The discrepancy between the target values and the identified material parameters is calculated by using the MAE. After five optimization runs, each sample was analyzed on the basis of the average error of all five found solutions. In all investigated samples, it is found that the convergence is successfully achieved in nearly all cases, and the objective function approaches zero with negligible deviation. An important observation was that the magnitude of the remaining error function after optimization showed a direct relationship with the quality of the identified material parameters. In the three detailed examples, a clear trend is visible that the scatter in the identified material parameters increases progressively as the value of the MAE increases; see Table 3, Table 4 and Table 5. All five solutions achieve an order of the residual error around 10^−5^, showing very good comparability of identified material parameters with original parameters. It is also noted that the optimal parameter set corresponding to the lowest error value showed the best agreement with the original parameters. This clearly confirms the choice of the objective function which shows a strong sensitivity to the material parameters.

**Figure 7:**
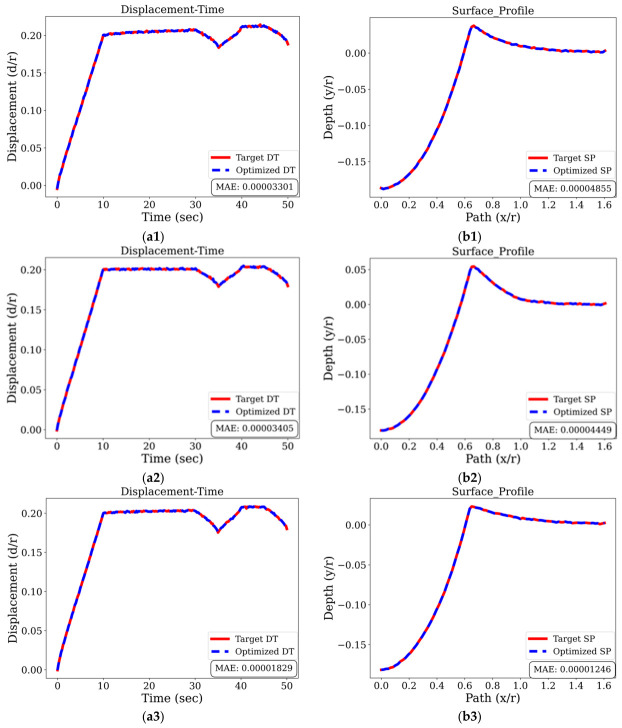
Optimization results of three selected reference materials: (**a1**), (**a2**), and (**a3**) show the optimized displacement–time curves for Sample 1, Sample 2, and Sample 3, respectively. (**b1**), (**b2**), and (**b3**) depict the optimized imprint profile for Sample 1, Sample 2, and Sample 3, respectively.

The comparative analysis of all three samples is performed based on the average error of all five obtained solution sets and their corresponding indentation response. Among the presented three examples, Sample 3 showed the lowest MAE of 3.63 × 10^−5^, which yielded excellent comparability to the reference parameters. In contrast, the other two samples 1 and 2 exhibited slightly larger errors (MAE) of 5.36 × 10^−5^ and 4.66 × 10^−5^, respectively, compared to the reference parameters. Figure 7 depicts the optimized DT curve and the IP in the case of three samples, which are reconstructed using the mean values of the identified material parameters obtained from five optimization runs. All three samples exhibit excellent fit between the reference and the optimized DT curve and IP with negligible error. Sample 3 depicts an excellent fit, showing the MAE of 1.829 × 10^−5^ and 1.246 × 10^−5^ for the DT curve and the IP, respectively; see Figure 7(a3,b3). Whereas a similar high-quality agreement is also found for Sample 1 and Sample 2, illustrated by Figure 7(a1,b1,a2,b2). Overall, in all cases, the value of the objective function remains very close to zero, depicting a very good fit of curves.

By following the same procedure of inverse parameter identification for twenty-eight independent samples, the robustness of the developed framework is further validated. In all cases, a reliable parameter recovery is observed when the magnitude of the residual error function approaches a value of less than 8 × 10^−5^. If the residual error is larger, the quality of the identified materials parameters is not guaranteed, and the optimization should be continued until a better approximation to the minimum of the error function is found. It is noted here that due to our proposed method, a minimum corresponding to the defined objective function value of zero is guaranteed to exist, as the optimization and the generated reference indentation responses are based on the same surrogate models. Across all tested samples for inverse parameter identification, the proposed inverse framework showed consistent and reliable performance. In 80% of cases, the objective function converged within the range of 1 × 10^−5^ to 7 × 10^−5^, showing a very close correspondence to the reference parameter sets with only slight deviations for some parameters.

Within this interval, the quality of identified parameters gradually improves as the error function decreases. In 10% of the test cases, the error function converges within the range of 1 × 10^−6^ to 9 × 10^−6^, yielding excellent comparability to the original parameters. For the remaining 10% samples, the optimization stabilized within 1 × 10^−4^ to 4 × 10^−4,^ exhibiting relatively larger deviation with reduced quality, particularly in the case of parameters Q and $C_{2}$. However, the quality of the identified parameters is significantly compromised if the magnitude of the residual exceeds 5 × 10^−4^.

In this case, the errors in individual parameters can be large, as the solution is not unique. These findings emphasize the necessity of the strict convergence criterion to improve the robustness of the material parameter identification. The present study indicates that reliable parameter identification requires convergence of the objective function below 10^−4^.

### 5.2. Verification of Obtained Parameters

**Figure 8:**
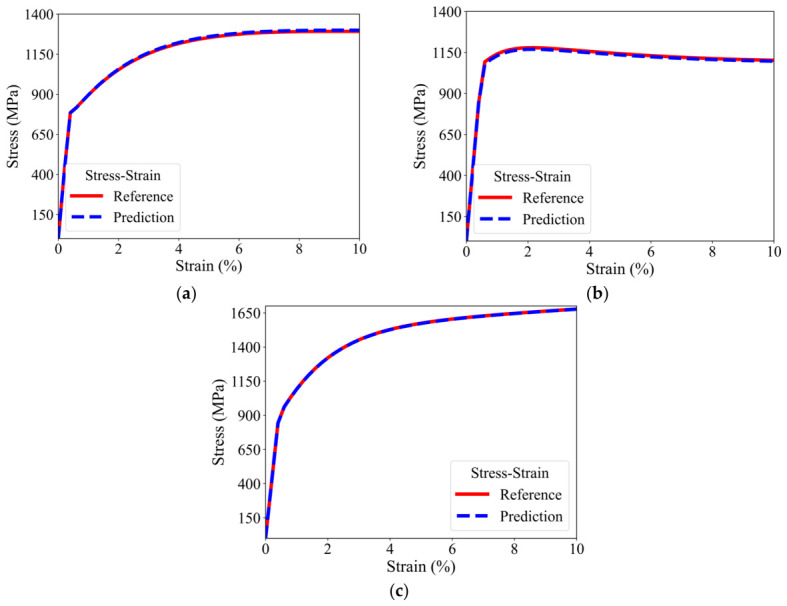
The comparison of uniaxial stress–strain curves by simulating the tensile test for original and identified material from (**a**) Sample 1 (**b**) Sample 2, and (**c**) Sample 3.

For the verification of the identified material parameters, the uniaxial tensile test is simulated to predict the stress–strain curve by using the mean identified material parameters for three selected samples described in Section 5.1. A comparison is drawn between the simulated stress–strain curve using reference parameters and the identified material parameters. Figure 8 illustrates a very close correspondence between the prediction and the reference stress–strain curve. Depending on the accuracy of the reproducibility of the parameters, all samples showed excellent prediction, particularly in the case of Sample 3; see Figure 8c.

### 5.3. Uniqueness Analysis

**Figure 9:**
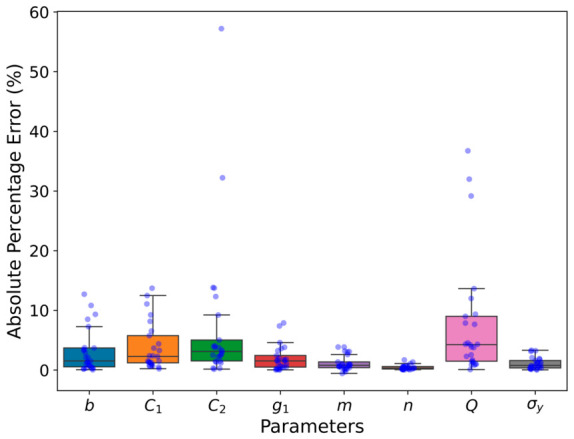
The box plot illustrates the distribution of absolute percentage error (%) with respect to each identified material parameter for all twenty-eight investigated reference datasets, while each blue dot depicts an individual investigated sample.

To evaluate the overall robustness and uniqueness of the identified material parameters a quantitative analysis is performed across all twenty-eight test cases. An absolute percentage error is measured between each reference parameter and the mean value of the five optimal solutions obtained from each case. The comparison of all identified material parameters ($\sigma_{y}$, $C_{1}$, $g_{1}$, $C_{2}$, Q, b, n, m) with respect to the absolute percentage error (%) is depicted by the box plot; see Figure 9. Each box demonstrates the distribution of statistical errors for one parameter, showing the median, interquartile range, and spread across twenty-eight cases. Each individual case is represented by the blue scattered points. The distribution of error percentage across the eight evaluated parameters varies in both central tendency and dispersion. The parameters n, m and $\sigma_{y}$ yielded the lowest median errors below 1%, with very narrow interquartile ranges. It demonstrates the strong consistency and minimal variability over all twenty-eight test cases. In particular, n emerged as the most stable parameter, showing the lowest spread and median error of (0.3–0.5%). In contrast, the parameters Q and $C_{2}$ revealed a higher median error of (4–5%) and 3%, respectively. Furthermore, the wider interquartile range and greater variability are observed particularly for parameter Q. These two parameters also showed a few upper outliers, with error values exceeding 30%. However, in both cases, the median error still remains within an acceptable range, and overall, the mean error is less than 7%. It is also noted that most of the scatter points lie below the median error. In the case of parameters Q and $C_{2}$, the wider error distributions can be attributed to their lower sensitivity to the indentation response under the applied loading conditions. The compensation between kinematic hardening and isotropic hardening can cause an indistinguishable indentation response for multiple parameter combinations, especially when the objective function does not converge to 10^−5^. It is evident in the case of outliers that the objective function converges to a relatively higher residual value, e.g., 10^−4^.

The parameters *b*, $C_{1}$, and $g_{1}$ demonstrate intermediate behaviour with median error ranging from 1% to 3%, showing a small spread and limited outliers, particularly in the case of parameter $g_{1}$. However, all these parameters demonstrate significantly lower variability as compared to parameters Q and $C_{2}$. Overall, Figure 9 demonstrates the statistical validation of the central claim of the present study. The developed inverse framework can reliably identify the eight unknown material parameters from the indentation response when the objective function reduces below 10^−4^, and the stability of the solution for the material parameters gradually improves with further reduction in the error function. In all twenty-eight cases of inverse analysis, most of the parameters are recovered with acceptable error. However, the parameters n, m, $\sigma_{y}$, and $g_{1}$ showed excellent comparability with the original parameters.

### 5.4. Sensitivity Analysis

**Figure 10:**
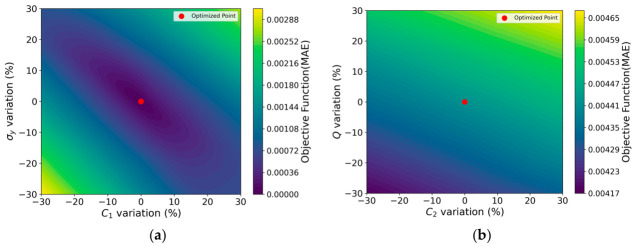
Normalized 2D objective function depicting sensitivity and coupling effect of (**a**) sensitive parameters $C_{1}$ and $\sigma_{y}$, and (**b**) less sensitive parameters $C_{2}$ and Q, evaluated around the optimal solution obtained from Sample 1.

The sensitivity of the objective function is verified around the optimal parameter set. Due to the similar results of inverse analysis across all twenty-eight samples, only one optimal set of parameters obtained from Sample 1 is analyzed in this section. The optimal solution contains eight identified parameters. Therefore, only four parameters ($C_{1}$, $\sigma_{y}$, $C_{2}$ and Q) are selected to show their sensitivity and coupling effect with respect to the designed objective function. In Figure 10, a normalized 2D objective function landscape is plotted by varying two parameters up to ±30% around the optimum, while keeping the other parameters fixed at their identified values. The contour shows the normalized objective function, and the red point depicts the optimized solution. Figure 10a demonstrates that the $C_{1}$ and $\sigma_{y}$ parameters exhibit sharp and well localized minima around the optimized point. It shows that the objective function sharply increases as $C_{1}$ and $\sigma_{y}$ slightly deviate from their optimum values. This behaviour illustrates that the parameters $C_{1}$ and $\sigma_{y}$ are highly sensitive to the indentation response. A slight variation in these parameters can produce a noticeable change in the indentation response. Consequently, these parameters showed high consistency and reproducibility in the inverse parameter identification. On the other hand, the 2D landscape for parameter Q and $C_{2}$ shows a broader valley with widely spaced contours; see Figure 10b.

The elongated valley indicates that the different combinations of Q and $C_{2}$ can produce a similar indentation response, which compromises the uniqueness of the identified material parameters. These plots illustrate that those parameters associated with a sharp and well-defined minimum can be identified with high fidelity, whereas the parameters showing flatter and elongated valleys are less constrained to a unique value. These plots validate the statistical findings that are shown by Figure 9 in Section 5.3. They also confirm that the convergence of the objective function to zero is essential for unique parameter identification.

## 6. Conclusions

The present study demonstrates a surrogate model-based approach for the inverse identification of material parameters from the micro-indentation data with reduced computational cost. Using artificial neural networks (ANNs), three forward surrogate models were trained on the FEM-based simulation data to predict the maximum indentation force, displacement–time curve, and the imprint profile as a function of the material parameters in a numerically efficient way. While the surrogate models for the displacement–time curve and imprint profile have a very high accuracy, the surrogate model for the maximum indentation force shows the highest relative residual error of all models, with a value of 0.0072. This can be attributed to the strong sensitivity of a single peak value in the region of sharp transition at the maximum loading stage where the behaviour of the material is strongly nonlinear. However, the residual error less than 1% is still acceptable for practical applications. For the validation of the proposed method, the inverse parameter identification is conducted for twenty-eight reference parameter sets that have not been used for training or testing. By applying the numerically efficient surrogate models in a genetic algorithm, eight unknown material parameters for initial yield strength, kinematic and isotropic hardening and creep ($\sigma_{y}$, $C_{1}$, $g_{1}$, $C_{2}$, Q, b, n, m), are identified with high accuracy. The uniqueness study revealed that the inclusion of the curve-based indentation response, i.e., displacement–time curve and imprint profile, in the objective function yields a good reproducibility of all identified material parameters. It is noted that the exclusion of maximum indentation force from the optimization even improved the uniqueness of identified material parameters. This behaviour can be attributed to the richer information provided by the curve-based targets and the scalar nature of the maximum force. Furthermore, an enhanced reproducibility of parameters was observed by giving the highest weights to the displacement–time curve in the error function. It is also evident that the uniqueness of the identified material parameters gradually improves as the error function approaches a value of zero. A useful indicator for a reliable and consistent identification of material parameters is that the error function has reached a value of less than 10^−4^. If the final error function has higher values, it is likely that the parameter identification is not yet completed properly. Among all identified material parameters, n, m, and $\sigma_{y}$ showed excellent agreement with reference parameters in all cases. In contrast, parameters Q and $C_{2}$ showed relatively non-consistent results, as there is only a low sensitivity of the indentation results on these parameters. The genetic algorithm showed its capability to explore the entire parameter space due to the efficiency of the surrogate models. The combination of both played a key role in parameter identification with stable and unique results. Based on these findings, the developed approach is found to be an efficient and robust alternative to computationally expensive FEM simulations and provides a good basis to investigate the inverse analysis for the experimental data.

## Figures and Tables

**Table 1:** Input material parameters for the data generation.

Parameters	Min	Max
E (GPa)	211	
$\sigma_{y}$ (MPa)	600	1200
$C_{1}$ (MPa)	5000	100,000
$C_{2}$ (MPa)	10	1000
$g_{1}$	1	200
$g_{2}$	0	
m	−0.9	−0.4
n	2.5	5.0
$q_{o}$ (MPa)	1000	
Q (MPa)	−500	500
b	0	15

**Table 2:** Comparison of FEM-based maximum indentation force and prediction by the developed surrogate model.

Sample	Maximum Indentation Force (f/r^2^) FEM Result	Maximum Indentation Force (f/r^2^) Surrogate Model	Absolute Relative Error
1	7484.1	7464.6	0.003
2	6226.6	6098.2	0.021
3	7256.5	7372.0	0.016

**Table 3:** List of five optimal solutions resulting from five independent optimization runs for Sample 1.

Reference Parameter	b 4.50	$C_{1}$ (MPa) 33,500.00	$C_{2}$ (MPa) 307.00	$g_{1}$ 60.70	m −0.75	n 3.25	Q (MPa) −200.0	$\sigma_{y}$ (MPa) 780.00	MAE 0
Solution 1	4.25	33,673.05	302.98	61.37	−0.75	3.26	−187.08	778.06	3.23 × 10^−5^
Solution 2	4.43	34,462.42	282.52	61.82	−0.75	3.27	−182.98	780.55	3.95 × 10^−5^
Solution 3	4.50	32,662.69	298.47	61.05	−0.74	3.26	−177.23	797.16	5.28 × 10^−5^
Solution 4	4.31	35,259.52	265.09	61.38	−0.77	3.24	−196.86	746.89	6.95 × 10^−5^
Solution 5	4.49	32,669.94	288.49	60.44	−0.72	3.27	−173.66	803.48	7.59 × 10^−5^
Mean	4.39	33,745.52	287.51	61.21	−0.74	3.26	−183.52	781.23	5.36 × 10^−5^

**Table 4:** List of five optimal solutions resulting from five independent optimization runs for Sample 2.

Reference Parameter	b 12.00	$C_{1}$ (MPa) 24,000.00	$C_{2}$ (MPa) 802.00	$g_{1}$ 160.20	m −0.80	n 4.50	Q (MPa) −300	$\sigma_{y}$ (MPa) 1080.00	MAE 0
Solution 1	11.94	24,234.02	800.46	160.03	−0.81	4.51	−299.41	1073.70	2.26 × 10^−5^
Solution 2	12.21	26,997.07	773.22	166.03	−0.80	4.53	−294.04	1062.84	4.74 × 10^−5^
Solution 3	11.90	26,003.81	770.61	165.29	−0.80	4.55	−285.54	1054.95	5.15 × 10^−5^
Solution 4	12.15	23,151.01	757.09	158.64	−0.79	4.53	−280.81	1073.90	5.41 × 10^−5^
Solution 5	11.91	24,187.30	755.78	161.38	−0.79	4.54	−277.46	1062.01	5.71 × 10^−5^
Mean	12.02	24,914.64	771.43	162.27	−0.80	4.53	−287.45	1065.48	4.66 × 10^−5^

**Table 5:** List of five optimal solutions resulting from five independent optimization runs for Sample 3.

Reference Parameter	b 10.43	$C_{1}$ (MPa) 50,106.26	$C_{2}$ (MPa) 737.39	$g_{1}$ 82.99	m −0.74	n 3.95	Q (MPa) 172.24	$\sigma_{y}$ (MPa) 906.98	MAE 0
Solution 1	10.09	50,267.62	735.93	84.53	−0.73	3.95	186.49	906.95	2.07 × 10^−5^
Solution 2	10.00	50,814.97	724.24	85.51	−0.74	3.94	192.25	902.08	2.68 × 10^−5^
Solution 3	10.91	49,550.28	721.24	79.14	−0.74	3.96	162.79	903.79	3.00 × 10^−5^
Solution 4	10.51	47,436.05	767.62	77.85	−0.73	3.96	158.23	918.55	4.95 × 10^−5^
Solution 5	10.33	53,508.84	723.92	89.40	−0.74	3.94	193.81	894.15	5.45 × 10^−5^
Mean	10.37	50,315.55	734.59	83.29	−0.74	3.95	178.71	905.10	3.63 × 10^−5^

## Data Availability

The original contributions presented in this study are included in the article. Further inquiries can be directed to the corresponding author.
